# Diagnosis and Treatment of Acute Pancreatitis

**DOI:** 10.3390/diagnostics12081974

**Published:** 2022-08-15

**Authors:** Julia Walkowska, Nicol Zielinska, R. Shane Tubbs, Michał Podgórski, Justyna Dłubek-Ruxer, Łukasz Olewnik

**Affiliations:** 1 Department of Anatomical Dissection and Donation, Medical University of Lodz, 90-752 Lodz, Poland; 2 Department of Neurosurgery, Tulane University School of Medicine, New Orleans, LA 70112, USA; 3 Department of Neurosurgery and Ochsner Neuroscience Institute, Ochsner Health System, New Orleans, LA 70112, USA; 4 Department of Anatomical Sciences, St. George’s University, St. George’s P.O. Box 7, Grenada; 5 Department of Neurology, Tulane University School of Medicine, New Orleans, LA 70112, USA; 6 Department of Structural and Cellular Biology, Tulane University School of Medicine, New Orleans, LA 70112, USA; 7 Department of Surgery, Tulane University School of Medicine, New Orleans, LA 70112, USA; 8 Department of Diagnostic Imaging and Interventional Radiology, 90-549 Lodz, Poland

**Keywords:** resonance magnetic, MRI, diagnosis, pancreas

## Abstract

The pancreas is a glandular organ that is responsible for the proper functioning of the digestive and endocrine systems, and therefore, it affects the condition of the entire body. Consequently, it is important to effectively diagnose and treat diseases of this organ. According to clinicians, pancreatitis—a common disease affecting the pancreas—is one of the most complicated and demanding diseases of the abdomen. The classification of pancreatitis is based on clinical, morphologic, and histologic criteria. Medical doctors distinguish, inter alia, acute pancreatitis (AP), the most common causes of which are gallstone migration and alcohol abuse. Effective diagnostic methods and the correct assessment of the severity of acute pancreatitis determine the selection of an appropriate treatment strategy and the prediction of the clinical course of the disease, thus preventing life-threatening complications and organ dysfunction or failure. This review collects and organizes recommendations and guidelines for the management of patients suffering from acute pancreatitis.

## 1. Introduction

Two phases of AP have been identified: early and late, while the severity has been divided into mild, moderate, and severe [1]. Basically, at least two of the following symptoms must be present for a diagnosis of acute pancreatitis: abdominal pain, described as a persistent and severe epigastric pain often radiating to the back with acute onset; activity of the serum lipase or amylase at least three times greater than the upper limit of normal; and the characteristic symptoms of acute pancreatitis detected by ultrasonography (US), computed tomography (CT), or magnetic resonance imaging (MRI) [2].

There are two classifications systems of AP: the Determinant-Based Classification of Acute Pancreatitis Severity (DBC) and the Revised Atlanta Classification 2012 (RAC). Patients who have persistent organ failure, categorized as severe AP, have the highest risk of death. Due to that, it is important to predict and diagnose an episode of severe AP [3]. Banks et al. [1] described the Atlanta classification and the definitions of acute pancreatitis (AP). In their opinion, the above-mentioned classification determines better communication between clinicians, standardizes the reporting of research results, and also introduces clear definitions that enable the classification of acute pancreatitis, using easily identifiable radiological and clinical criteria.

The etiology of AP should be determined on admission. Early initiation of diagnostics to determine the etiology increases the probability of stating a proper diagnosis. Moreover, it enables the implementation of appropriate treatment and methods to prevent complications and allows the taking of measures to prevent subsequent attacks of pancreatitis. The etiology is defined on the basis of a detailed personal and family history of pancreatic disease, physical examination, laboratory serum tests, and imaging. Another measure that should be taken on admission is to predict the outcome of the AP. It is advised to evaluate host risk factors, clinical risk, and response to initial therapy [4].

To predict the severity and mortality of AP, clinical data (including assessment of organ function) are assessed, laboratory tests and imaging are performed, and severity-of-the-disease rating systems are used. Those measures should be taken on admission and at 48 h [3,4].

The management of the patient is based on the providing of supportive care, including, inter alia (in. al.), fluid resuscitation, pain control, and organ function assessment; ensuring adequate nutrition; and providing interventional treatments, such as cholecystectomy or endoscopic sphincterotomy, or necrosectomy in the case of necrotizing pancreatitis [3,4].

This paper describes the available diagnostic methods used to diagnose AP and assess the subtype of AP, which is the severity of AP, and to present the management of the patient suffering from AP.

## 2. Classification of Acute Pancreatitis

Performing specific diagnostic measures should be adjusted to the presumed cause and condition of the patient. Firstly, the patient has to meet two of the three criteria to be diagnosed with AP. Subsequently, local complications are assessed using diagnostic imaging methods, and systemic complications are evaluated based on the assessment of the efficiency of the respiratory, cardiovascular, and urinary systems. These complications determine the severity of the AP, which affects the management with the patient [1,5].

AP is diagnosed on the basis of two of three criteria—typically belt-like abdominal pain, an elevated serum lipase level three times above the normal threshold, and radiological imaging signs of pancreatitis [4,5,6]. The first two are present in the most of patients, whereas the latter occurs slightly less frequently. Due to that, in the vast majority of cases, diagnosis of AP can already be established on the basis of abdominal pain and an elevation of pancreatic enzymes [6].

The revised Atlanta classification system from 2012 [5], defining the clinical diagnosis, CT manifestations, and the disease course of acute pancreatitis, distinguishes two morphologic subtypes of AP: interstitial oedematous pancreatitis and necrotizing pancreatitis.

The aforementioned classification, evaluating additional local or systemic complications as well as the presence and duration of organ failure, divides AP into three subtypes: mild AP, moderately severe AP, and severe AP [3,5]. The mortality is different among the subtypes of AP. For instance, severe, necrotizing AP leads to a mortality rate reaching 25%, while mild, edematous AP includes a mortality of only 1%. Twenty to thirty percent of patients suffering from AP experience recurrent pancreatitis attacks, and of these, 10% develop CP [7,8,9].

Pancreatitis can lead to local or systemic complications. Each of those has its own characteristics based on the patient’s symptoms. AP can be a mild, self-limiting disease that requires only supportive measures, but it also might turn into a severe disorder with life-threatening complications, such as insufficiency of the respiratory and cardiovascular systems or kidney failure [2,5]. The characteristics of the aforementioned complications and the subtypes of acute pancreatitis are described in the table below (Table 1).

The RAC and DBC are similar in determining the diagnosis and severity of AP. The RAC, in addition to the severity classification, clearly defines the definition of AP, highlights the onset of pain, and defines individual local complications, as well as interstitial and necrotizing pancreatitis [1,3]. The RAC has three categories: mild, moderately severe, and severe. The DBC added a fourth category—critical, based on two major determinants of mortality: organ failure and (peri)pancreatic necrosis. Persistent organ failure with infected necrosis is associated with the highest risk of death. Therefore, those patients should be admitted to an intensive care unit and constantly monitored. Accordingly, the diagnosis and prediction of severe AP is crucial, as is the identification of the patients with a high risk of developing complications [3].

## 3. Diagnosis of Acute Pancreatitis

According to the recommendations, the diagnosis of acute pancreatitis is based on blood tests to determine the level of serum lipase and amylase and imaging techniques: magnetic resonance cholangiopancreatography (MRCP), CT, and US [2]. Measurements of serum and urinary enzymes are used to diagnose the AP, none of them allow the evaluation of the severity of the AP and the accurate prediction of the clinical course of the disease [10,11,12].

Nowadays, due to technological development, radiologic imaging plays a more and more significant role in the management of the patient. The above-mentioned imaging techniques provide crucial information for the diagnosis and the course of the disease. Specifically, ultrasonography is recommended as a first and basic imaging test performed in patients with suspected AP in order to confirm or exclude the diagnosis as well as detect the possible cause of the disease, while MRI and CT are useful in diagnosing local complications and discovering the necrosis of the pancreas or in assessing the severity of the AP. The latter two have specific indications; they are performed to broaden the diagnosis or when ultrasound does not visualize the structures properly, making it impossible to make an unequivocal diagnosis [13]. It is worth emphasizing that the diagnosis of AP is as important as the diagnosis of the etiology of AP, which in many cases is associated with an inadequate workup.

### 3.1. Laboratory Test and Indicator Enzymes

People suffering from the acute onset of a persistent, diffuse abdominal pain or acute epigastric pain should be diagnosed for acute pancreatitis. Therefore, it is important to know the diagnostic accuracy of serum lipase, serum amylase, urinary trypsinogen-2, and urinary amylase, either alone or in combination, for the diagnosis of AP [14].

The accurate diagnosis of AP, the early assessment of the severity of AP, and the identification of the etiology are criteria that should be met by an ideal laboratory test in assessing the condition of a patient with AP. Currently, no biochemical test has been identified that fulfils the above-mentioned criteria and can be considered the “gold standard” for the diagnosis and evaluation of the severity of AP [10].

Nonetheless, the relevant and currently commonly used laboratory tests in the diagnosis of AP are serum lipase and serum amylase [10]. Based on multiple studies, lipase serum has been found to be a more reliable indicator of AP than serum amylase, whereas a reliable early diagnosis of AP is assured by urinary strip tests for trypsinogen-2 and trypsinogen activation peptide (TAP) [15]. Other enzymes used in the diagnosis of AP, such as pancreatic isoamylase, immunoreactive trypsin, chymotrypsin, or elastase, are not better than lipase; furthermore, they are more inconvenient and expensive. Measurement of the levels of the aforementioned enzymes should be reserved for situations of uncertain diagnosis. Neither enzyme assay is associated with the severity of AP and cannot precisely predict the consecutive clinical course of the patient [10,11,12]. According to Al-Bahrani and Ammori [15], a biliary etiology is reliably predicted by early transient hypertransaminasemia, whereas a reliable predictor of alcoholic etiology is serum carbohydrate-deficient transferrin [15]. Urinary enzymes are less significant in clinical practice than serum enzymes among the adults. However, urinary enzymes can be used in the case of AP in children [16]. Nevertheless, it is worth knowing all the available diagnostic methods.

### 3.2. Laboratory Tests

#### 3.2.1. Serum Lipase and Amylase

According to a clinical practice guideline, published in 2016 by Greenberg et al. [2,17], concerning the management of acute pancreatitis, in all patients with suspicion of acute pancreatitis a level of serum lipase should be tested because of its slightly higher (79%) sensitivity in comparison with other serum and urine tests (72%). The diagnosis of acute pancreatitis is made when a serum lipase activity is at least three times greater than the upper limit of normal.

A serum amylase test is also performed in the diagnostics of AP, but it has a lower clinical value. The key blood biochemical parameter in the detection of acute pancreatitis is a serum lipase, which is characterized by an earlier and longer-lasting elevation than a serum amylase. Specifically, the lipase level generally stays elevated for up to two weeks, while the amylase level is elevated for up to five days [2,18].

Additionally, a serum lipase test has a slightly higher sensitivity compared to the amylase test. At day 0–1 from the onset of symptoms, 100% is reached for lipase, while it is 95% for amylase. For day 2–3, the sensitivity ranges from 85%, whereas the specificity approximates 82% for lipase, in comparison to 68% for amylase. Based on the presented results, it can be concluded that lipase is particularly useful in the case of a delay between the time the patient seeks medical attention and onset of the symptoms [2,18].

As reported by The American College of Gastroenterology in 2013, the measurement of both serum lipase and serum amylase does not demonstrate advantages in either treatment or profitability [2,18]. Additionally, serum lipase has been found to be more sensitive than serum amylase among patients with acute pancreatitis secondary to alcohol abuse [1,2,19]. Another research performed by Gwozdz et al. [20], presents the diagnostic values of serum and urine enzyme assays in the recognition of AP. The study compares the diagnostic sensitivities of serum lipase, amylase, trypsinogen, elastase-1, the 2 h-timed urine amylase excretion, and the clearances of amylase and creatinine. All the serum tests showed the same sensitivity at the time of admission; however, in the following days, the serum lipase, trypsinogen, and elastase-1 tests presented considerably higher sensitivity than the serum amylase assay. During the second and following days, the diagnostic value of timed urine amylase excretion did not predominate over the serum amylase, and the ratio of amylase and creatinine clearances completely did not differ from each other [20].

The study by Rompianesi et al. [18], compared the diagnostic exactness of serum lipase, serum amylase, urinary amylase, and urinary trypsinogen-2 in the diagnosis of AP. Serum lipase and serum amylase, with a more than three times greater value than the standard threshold level, and urinary trypsinogen-2, with a value higher than the threshold of 50 ng/mL, seem to have similar sensitivities (79%, 72%, and 72%, respectively) and specificities (89%, 93%, and 90%, respectively). Researchers suggest that one of the above-mentioned parameters should have a low threshold, which would allow the initiation of treatment for AP even when the other parameters are normal. Additionally, they conclude that the occurrence of other disease entities should be considered, despite the incorrect results of the above-mentioned parameters, in order to avoid a misdiagnosis of AP [17].

In conclusion, the biochemical diagnostic assay with a slightly greater clinical value is serum lipase. Lipase assays are nowadays instant, reliable, practical, more specific, and sensitive, and their price does not significantly exceed the price of amylase assays. The main advantages of serum lipase are the maintenance of elevated levels for a longer time in comparison with amylase, which is used in the case of the patients who initially present to the emergency department a few days after the onset of AP symptoms, and greater sensitivity in AP caused by alcohol overuse. The serum amylase assay is also performed in the diagnosis of AP, but it has a lower clinical value due to its greatest disadvantage, which is overall low specificity. A normal serum amylase should usually rule out the diagnosis of AP, except for AP secondary to hyperlipidemia and acute exacerbation of chronic pancreatitis, and when the assessment of amylase is delayed in the course of the disease. Nevertheless, the assessment of serum amylase has some advantages, such as inexpensiveness, ready availability, simple automated methods, and high sensitivity [10,12,21]. The measurement of serum lipase is not affected by hypertriglyceridemia, but some drugs (for instance furosemide) give a possibility of increasing the serum activity [22]. Other causes of increase are renal insufficiency, chronic pancreatitis, acute cholecystitis, or bowel obstruction [18,22], whereas the measurement of serum amylase is competitively interfered with by hypertriglyceridemia; so, falsely low results can be produced, but it can be modified by using lipid clearing agents [10,18]. Rarely seen abnormally low levels of amylase can occur during chronic pancreatitis, cystic fibrosis, smoking, obesity, and diabetes mellitus [18]. 

To summarize, the crucial information concerning the serum lipase and amylase, have been presented in the table below (Table 2).

#### 3.2.2. Urinary Trypsinogen-2

The sensitivity and specificity of the urinary trypsinogen-2 dipstick test are higher than those of the urinary amylase dipstick test [23,24]. Higher levels in the urine than in the serum can occur because of the breakdown of the protein and the release of peptides during the increased proteolytic activity in AP and the consecutive decrease in the ability of the renal tubuli to reabsorb proteins [25].

Urinary trypsinogen-2 remains increased for longer in patients with acute pancreatitis, compared to amylase, both in serum and urine [25]. It is also better in the differentiation between severe and mild AP than the serum and urine amylase [26].

A negative test excludes AP with a higher probability; therefore, it is more suitable for the screening of acute pancreatitis than serum lipase because of its higher sensitivity in comparison to serum lipase [25,27].

Yasuda et al. [28] confirmed that the rapid urinary trypsinogen-2 dipstick test and the levels of urinary trypsinogen-2 and TAP concentration may be considered as useful prognostic markers for the diagnosis of acute pancreatitis. The levels of urinary trypsinogen-2 TAP were considerably higher in patients with extended extra-pancreatic inflammation as evaluated by CT Grade, but not considerably higher in patients with hypo-enhanced pancreas lesions. Therefore, the measurement of urinary trypsinogen-2 and TAP could not select the patients who should have a CT examination [28].

#### 3.2.3. Urinary Amylase

The level of urinary amylase is measured by a clean-catch (midstream) urine sample or 24 h urine collection [17]. Urinary amylase presents lower values of sensitivity (83%) and specificity (88%) than serum amylase (85% and 91%, respectively) [25]. The urine test strips for amylase are considered to be useful bedside tests for the diagnosis of AP in patients with clinical pancreatitis [29].

The use of the urine level of amylase (uAm) is limited in practice because the diagnostic ability of uAm is inferior to that of the serum level of amylase (sAm). uAm has been used as a marker after endoscopic retrograde cholangiopancreatography or pancreas transplantation. The amylase creatinine clearance ratio (ACCR) is an index that uses uAm. ACCR is known to increase during pancreatitis. However, it has little diagnostic value because of its low specificity and sensitivity. Terui et al. [30] reported that the uAm/uCr ratio was correlated with sAm and may be an alternative to sAm for the prediction of hyperamylasemia. Furthermore, the correlation between sAm and uAm/uCr was low in babies and was significant in infants and schoolchildren. This indicated that the level of amylase itself cannot be used in babies. uAm/uCr could be appropriate for varied conditions of hyperamylasemia after the first year of life and does not appear to be influenced by elevated sCr. In the management of hyperamylasemia, uAm/uCr can potentially be used not for diagnosis but as a marker for following up on the levels of amylase. This result suggests the potential use of uAm/uCr as an alternative for sAm. Additionally, the use of urine samples results in a decreased need for blood sampling, which is especially beneficial in pediatric patients, and it reduces the risk of complications related to intravenous cannulation [30].

### 3.3. Ultrasonography

Approximately 70–80% of cases of AP are caused by gallstones and alcohol abuse. Due to the distinctions in management, the differentiation of the above-mentioned etiologies is significant. Ultrasonography is the first and basic imaging test performed in all patients with suspected acute pancreatitis because of its accessibility and low expense and because it gives no exposure to radiation [31]. This imaging modality is also used to diagnose acute biliary pancreatitis, excluding alcohol overuse as a main cause of AP. It allows the assessment of the condition of the biliary tract and the detection of biliary stones in the common bile duct (CBD).

The detection of gallstones by ultrasound has a sensitivity and specificity greater than 95% [2,32,33,34,35,36,37]. Other studies report on sensitivity reaching approximately 92–96% [38,39]. Nevertheless, ultrasound examinations are prone to some limitations that can be overcome by CT scans, such as the influence of intestinal gas occurring during ileus with bowel distension—a symptom commonly developing in the course of acute pancreatitis [2,32,33,34,35,36,37]. There are the ultrasonographic examination methods, which are recommended for imaging the pancreas. These are the grayscale examination, harmonic imaging, color doppler, and the power or spectral doppler. The sections used to depict the pancreas are transverse and longitudinal upper epigastric sections and, particularly for the head and tail of the pancreas, oblique intercostal and subcostal sections. Depending on the position of the transducer, the pancreas can be examined through sections with different locations relative to the stomach [40].

Specifically, positioning the transducer approximately halfway between the xiphoid appendix and the umbilicus provides examination through the transgastric or subgastric sections, while when the transducer is localized high in the epigastrum, the pancreas is visualized through the sections passing above the stomach antrum [40].

However, high epigastric sections avoiding the colon (thus decreasing the risk of visualizing the intestinal gas) and transgastric sections, as well as sections using the left lobe of the liver as an acoustic window, are considered to be the best ultrasound windows that can be obtained during ultrasonographic examination of the pancreas [40]. If the general condition of the patient with suspected AP allows it, the patient has to fast for at least 7–8 h, which is necessary to perform an appropriate ultrasound examination of the pancreas. The occurrence of food mass in the stomach might prevent the performance of precise and complete imaging of the pancreas and might also create images falsely suggesting pancreatic tumors [40].

Therefore, if it is necessary to conduct a quick diagnosis without proper preparation of the patient, for instance when the management of the patient is urgent and has to be immediate, the images obtained with the use of ultrasound may be of poor quality and thus provide an uncertain diagnosis. As mentioned above, US of the abdomen provides detection of the gallstones with high sensitivity. However, the sensitivity decreases significantly, to the level of 65%, if the gallstones are localized in the infundibulum of the gallbladder or the diameter of the stones is less than 3 mm [40].

**Figure 1:**
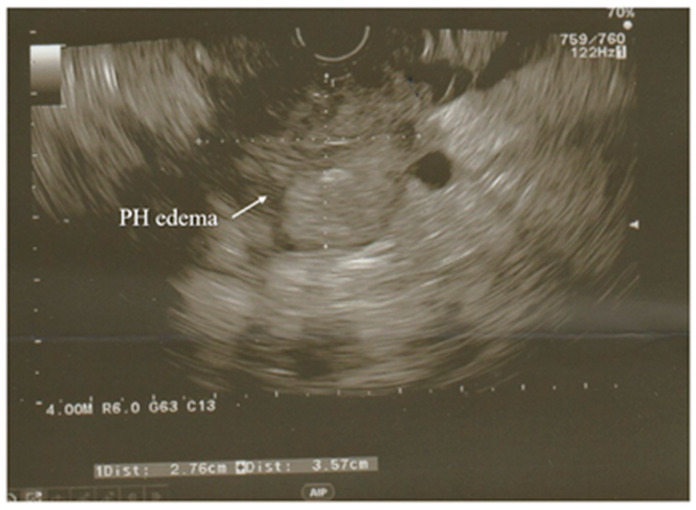
Acute pancreatitis with hypoechoic enlarged pancreatic head seen in EUS examination. PH—head of the pancreas.

**Figure 2:**
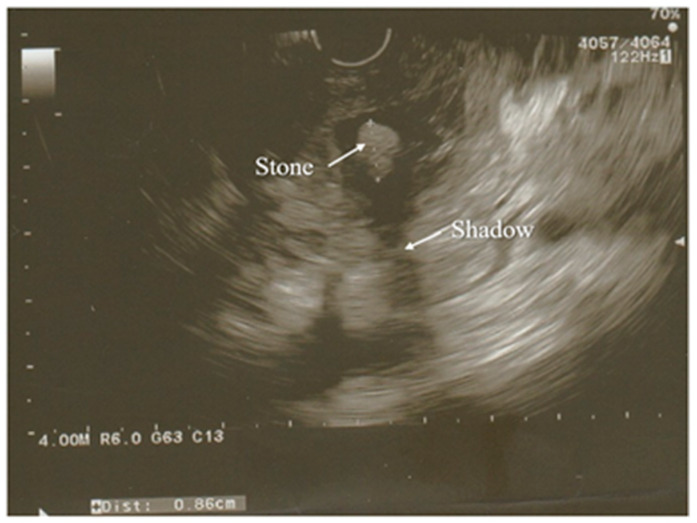
Partially calcified gallstone (poor shadow behind it) was seen in the distal part of a common bile duct during the EUS examination. Surrounding pancreatic parenchyma is edematous.

A modification of the standard ultrasound examination, with minimal invasiveness and high accuracy, used by choice to investigate the pancreas and biliary tract, is endoscopic ultrasound (EUS). Exemplary images from EUS examination have been presented on the Figure 1 and Figure 2. According to the results of the prospective study, EUS detects gallstones with a higher sensitivity in comparison to US (100% and 84%, respectively). Moreover, EUS is better than US in imaging the gallbladder because of the close proximity to the biliary system and, resulting from this, its high-image resolution [38]. Similarly, EUS provides improved spatial resolution in comparison to the MRI and CT scan, also owing to the closeness of the EUS probe to the pancreas [41]. In addition, EUS is a minimally invasive diagnostic modality and does not exhibit a comparatively high complication rate, unlike the ERCP. Both the EUS and the ERCP are characterized by the sensitivity reaching 97% for diagnosing choledocholithiasis. Due to this, EUS is helpful in the selection of the AP patients requiring therapeutic ERCP and thus avoiding the complications associated with diagnostic ERCP [38,39,41]. Because of these advantages, EUS has become a valid imaging technique useful in the assessment of patients with pancreaticobiliary disease, including AP.

In the case of AP, EUS is used to establish the cause of the disease after the decline of the acute attack of pancreatitis, while its usage during hospitalization for AP seems to be uncommon [38,42]. 

One of the diagnostic challenges for gastroenterologists is idiopathic acute pancreatitis (IAP) and idiopathic recurrent acute pancreatitis (IRAP). If the initial evaluation allows the recognition of the etiology of pancreatitis, as happens in 70–90% of cases, the patient is diagnosed with AP/RAP, while in the remaining 10–30% of the patients the etiology cannot be identified after the initial evaluation, which enables AP/RAP to be defined as IAP/IRAP [38,43,44]. The recognition of the etiology of pancreatitis is crucial for performing the appropriate evaluation, providing early treatment, and preventing relapse. Additionally, the determination of the etiology is important because 50% of untreated patients diagnosed with IRAP experience recurrent episodes that cause the progression of IRAP to CP [38,43].

There are different explanations for the situations when the etiology of AP cannot be determined. These are, for instance, the occurrence of biological abnormalities in the first days of AP, which cause lipid- or calcium-metabolism disturbances to be difficult to diagnose; microlithiasis, which is difficult to diagnose by the use of standard imaging modalities; and the inflammation or necrosis of the pancreas, which may disturb imaging of pancreatic cystic or solid tumors [38,45]. Management based on EUS is regarded as a reasonable approach for the assessment of patients with IAP/IRAP. The biliary tract disease is the most frequent diagnosis by EUS in IAP [38]. The most significant indications for EUS are suspicion of gallstones in the CBD and/or gallbladder and microlithiasis, while the most valid indication for EUS in AP is the suspicion of acute biliary pancreatitis when transabdominal ultrasound and tomographic examinations fail to depict biliary calculi. Although EUS allows the imaging of the entire gallbladder, pancreas, and biliary ductal system in AP in most cases, patients with severe pancreatic necrosis, variations of gastroduodenal anatomy, or a rare location of the gallbladder may cause occasional difficulties in performing the examination by the usage of EUS [38]. 

### 3.4. Magnetic Resonance Imaging

MRI and MRCP are used for the non-invasive evaluation of the pancreatic and biliary ducts, particularly the distal bile duct, which is hard to visualize by ultrasound, and are helpful in diagnosing the etiology of AP. MRI has many advantages, such as no exposure to radiation and the subsequent adverse effects on the human body; no use of a contrast agent in non-enhanced images; no premedication; no risk of developing complications; and the possibility to use it during an acute attack of pancreatitis and cholangitis, and it allows the visualization of the extraductal structures due to usage of standard T1-T2-weighted images. The non-enhanced MRI provides a clear presentation of the area of necrosis, and it is safe for the patients in the case of the impossibility of receiving iodinated contrast material due to kidney failure or allergies. Furthermore, MRI can visibly present local complications and stage the AP. Moreover, by the use of heavily T2-weighted sequences in non-enhanced MRCP, owing to high sensitivity to liquid, even a small amount of fluid in mild pancreatitis can be depicted [42]. MRI, together with MRCP, is used to image noninvasively a few fat or necrotic materials localized in a fluid-filled lesion and pancreatic duct system, which in turn allows the assessment of the duct integrity and of whether the collections surrounding the pancreas are in communication with the pancreatic ducts [36,37].

Sun et al. [36] reported that MRI without enhancement is more precise and reliable in assessing the severity of AP in comparison with CT [36]. Moreover, compared with CT, MRI is characterized by better soft-tissue contrast and the aforementioned no risk of radiation, which is significant for patients with AP requiring numerous follow-up examinations. Additionally, a non-enhanced MRI is a better radiologic modality in the diagnosis of mild AP as opposed to the CT [36].

The diagnosis of AP by the usage of an MRI is dependent on the occurrence of morphologic and peripancreatic changes. An MRI allows a good performance of an examination detecting pancreatic necrosis and the complications of AP, such as abscesses, pseudocysts, or hemorrhage [28]. Due to the increasingly common use of new MRI techniques that provide the diagnosis of pathological conditions associated with AP, the selection of management has improved. Additionally, multimodal MR images integrating a series of sequences, namely MRCP, T1- and T2-weighted imaging, dynamic contrast-enhanced (DCE) MR imaging, and diffusion-weighted imaging (DWI) with an apparent diffusion coefficient (ADC) map, are used more frequently for assessing AP before creating the plan of treatment [42]. This imaging technique is recommended only for patients whose CBD is not appropriately visualized or present as normal features in an ultrasound examination and in whom an elevation of liver enzymes is detected.

The meta-analysis by Romagnuolo et al. [46] presented the values of sensitivity and specificity of MRCP in the diagnosis of biliary obstruction, a probable pathomechanism of AP, which are, respectively, 95% and 97%. However, despite the advantages of MRCP, the cost of the examination limits its use in the diagnosis of gallstones, especially with the accessibility and usefulness of ultrasonography performed with the same objective [2,47].

### 3.5. Computed Tomography

**Figure 3:**
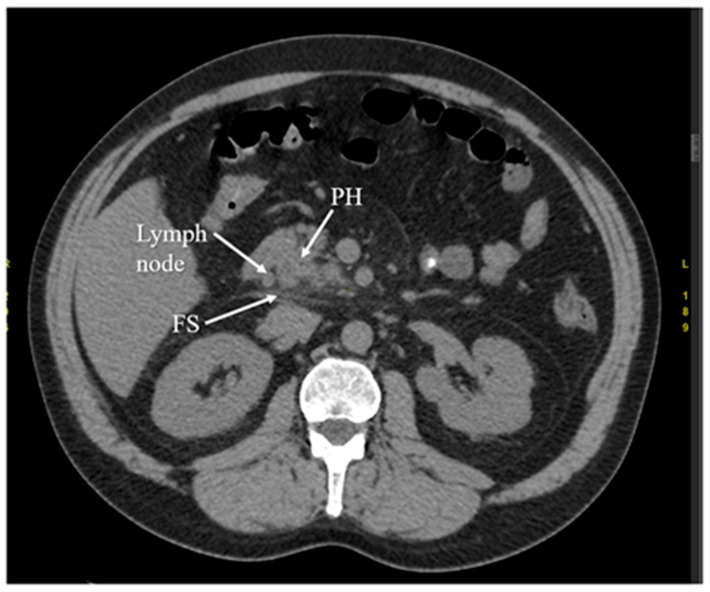
Early phase of the acute pancreatitis with stranding of surrounding fat (FS) and single, enlarged lymph node. The arterial phase of CT. PH—head of the pancreas.

**Figure 4:**
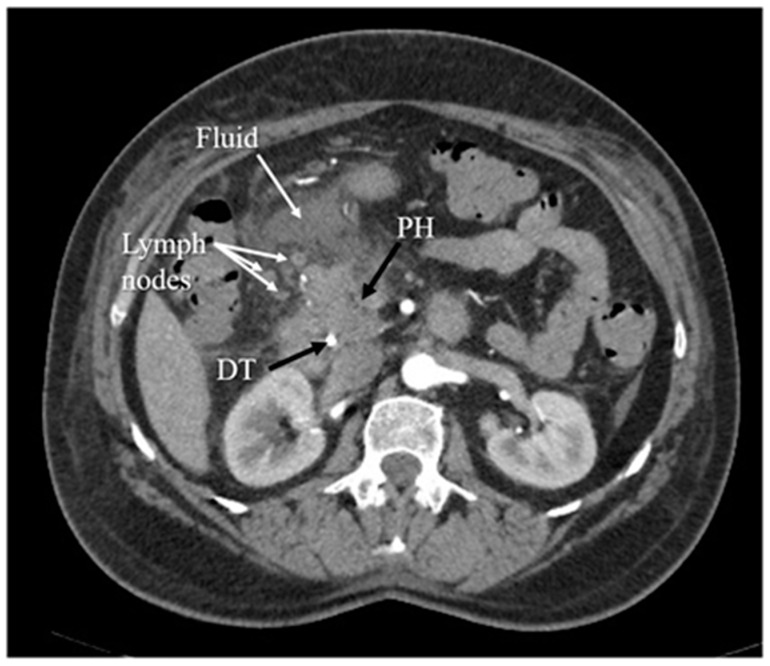
Inflammation of the head of the pancreas, with surrounding fluid and several enlarged lymph nodes. The arterial phase of CT. PH—head of the pancreas, DT—duodenal tube.

**Figure 5:**
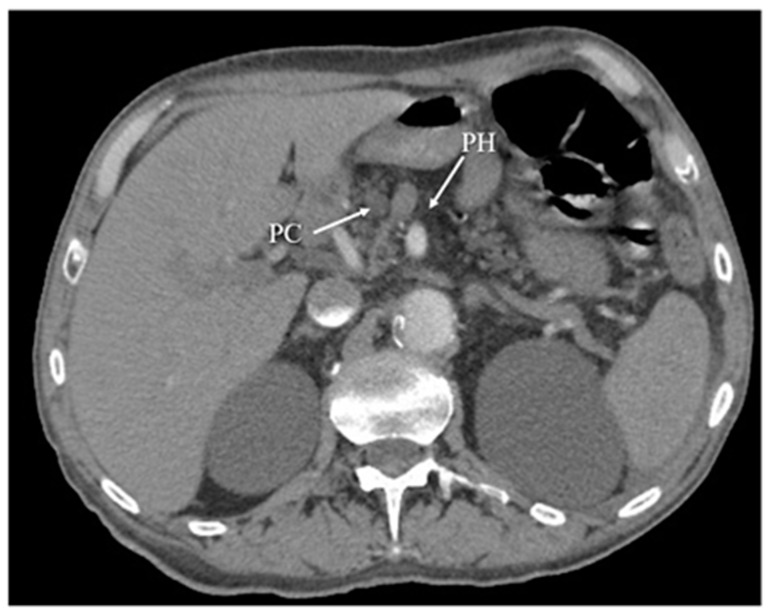
Chronic pancreatitis, with atrophy of pancreatic head (PH) parenchyma and pseudocyst (PC) in this region. The arterial phase of CT.

**Figure 6:**
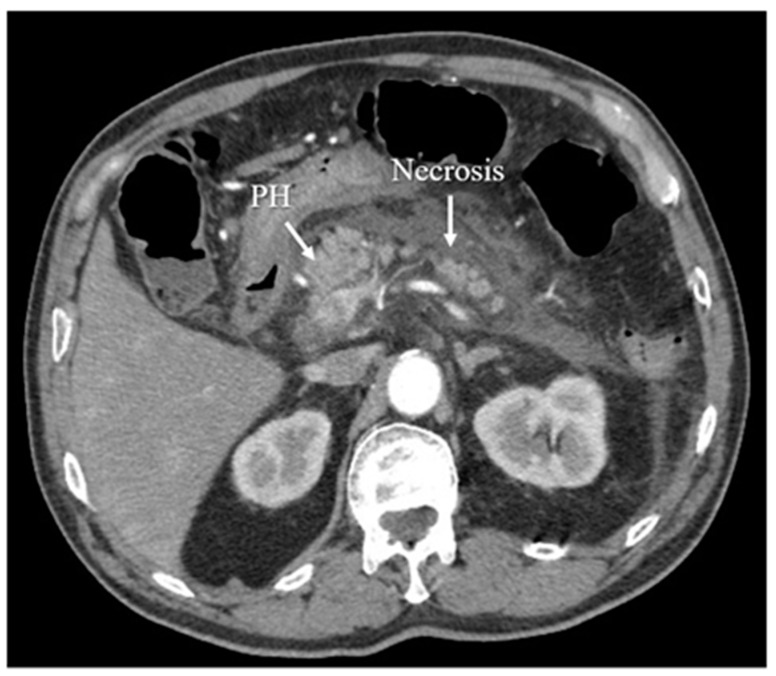
Acute necrotizing pancreatitis in the arterial phase of CT.

Another diagnostic method of acute pancreatitis is CT. Images obtained from CT have been shown in Figure 3, Figure 4, Figure 5 and Figure 6. CT is performed in order to evaluate the extent of AP and to assess complications. It is also regarded as a splendid diagnostic method, characterized by fast scans with high spatial resolution, used for discovering the necrosis of the pancreas, presenting local complications, grading the acuity of inflammation, and assessing the severity of AP [36,37,48]. CT also provides essential information for percutaneous management [13]. It should be performed selectively in two cases. The first includes patients with suspected local complications of acute pancreatitis, such as signs of shock, peritonitis, or ambiguous ultrasound results, whereas the second case concerns patients with severe abdominal pain and extensive differential diagnosis, which confirms acute pancreatitis [2]. In conclusion, each patient with abdominal pain and laboratory tests indicating AP should have a CT scan, in particular those patients with complications of AP or when the US examination is ambiguous.

CT presents higher accuracy and sensitivity than US in diagnosing and providing the extent of the disease [48]. However, it has disadvantages, such as a difficulty in distinguishing small quantities of necrotic or fat debris within one collection; it is also a potential radiation risk in the case of numerous follow-up scans [36,37]. Computed tomography performed in order to diagnose local complications is most valuable 48–72 h after the onset of symptoms rather than at the time of admission. If the patient’s normovolemia has been restored and fluids have been resuscitated appropriately, in the absence of contraindications (e.g., renal failure), the patient should receive an intravenous contrast in order to evaluate for pancreatic necrosis.

In advanced cases, CT is used to exclude local complications and distinguish necrotizing acute pancreatitis and interstitial acute pancreatitis. In this case, computed tomography has a limited use during admission because the above-mentioned differentiation in acute pancreatitis is typically possible more than 3–4 days from the onset of symptoms. However, CT finds its use in early diagnosis in the case of the broad differential diagnosis that must be narrowed [2,49,50]. According to the UK guideline for the management of acute pancreatitis, the only indications for the CT scan are inconclusive biochemical and clinical features [51].

To conclude, we have presented the advantages and disadvantages of radiological tests used in acute pancreatitis in the Table 3.

## 4. Etiology of Acute Pancreatitis

The etiology of acute pancreatitis should be determined on admission based on detailed personal history (i.e., previous acute pancreatitis, known gallstone disease, alcohol intake, medication and drug intake, known hyperlipidemia, trauma, and recent invasive procedures such as ERCP) and family history of pancreatic disease, physical examination, laboratory serum tests (i.e., liver enzymes, calcium, and triglycerides), and imaging (i.e., right upper quadrant ultrasonography) [4].

The most common causes of AP are gallstones and alcohol abuse. Therefore, transabdominal ultrasound and alcohol-use history should be obtained on admission to determine the etiology in all patients presenting with symptoms of AP because the treatment and follow-up are dependent on the etiology of pancreatitis. For instance, patients with biliary pancreatitis should undergo cholecystectomy, while in the case of alcoholic pancreatitis patients should attend dedicated follow-up visits to prevent recurrence [4,52,53,54]. Ultrasonography should be performed to evaluate the biliary tract and to determine whether the gallstones are present in the CBD. Furthermore, MRCP is recommended only in the case of the elevation of liver enzymes and inadequate imaging of CBD by the use of ultrasound [2]. Alcohol-induced AP is considered to require > 50 g of alcohol per day, while only approximately 5% of chronic alcoholics develop AP [53,55,56].

In the absence of gallstones or a history of alcohol abuse, a serum triglyceride level should be measured and considered as the cause of AP if the result is > 1000 mg/dl [57,58]. In drug-induced pancreatitis, the best-known medicaments are 6-mercaptopurine or azathioprine, isoniazid, loop diuretics, and didanosine [59,60]. Another cause of AP, though rare, that should be considered in patients aged 40 or more without another obvious etiology is pancreatic tumor or cystic neoplasm [53]. Post-ERCP pancreatitis is the most common serious adverse event attributed to the procedure, though there is debate over how to define this entity. It is defined as pancreatitis with the presence of new or worsened abdominal pain and amylase at least three times the normal at more than 24 h after the procedure, requiring admission or prolongation of planned admission to 2–3 days [61,62]. A unique form of chronic pancreatitis is called autoimmune pancreatitis (AIP), in which autoimmunity against unidentified auto antigens is responsible for the chronic fibro-inflammatory responses in the pancreas [63,64]. AIP is classified into type 1 AIP and type 2 AIP on the basis of clinical features, pathological findings, and IgG4 antibody (Ab) responses [65]. In general, it is accepted that type 1 AIP is a pancreatic manifestation of the systemic IgG4-related disease (IgG4-RD). IgG4-RD is a newly defined disease characterized by elevated serum levels of IgG4 Ab and the involvement of multiple organs. The predominant pathological feature of IgG4-RD, as well as type 1 AIP, is a massive infiltration of IgG4-expressing plasmacytes into the affected organs, accompanied by storiform fibrosis [66,67]. The presence of neutrophils, but not IgG4-expressing plasmacytes, is a pathological characteristic of type 2 AIP [68,69]. Eventually, idiopathic AP is defined as pancreatitis, with no etiology established after initial laboratory tests, transabdominal ultrasound, and CT [70].

In patients considered to have idiopathic AP, biliary etiology should be excluded by at least two US examinations and, if needed, MRCP and/or EUS to prevent recurrent pancreatitis. The subsequent step in diagnostics is EUS, performed after the acute phase of pancreatitis in order to assess for occult microlithiasis, neoplasms, or chronic pancreatitis. If the EUS is negative, MRCP is recommended as the following step to identify rare morphologic abnormalities. Additionally, CT of the abdomen should be performed. In the case of unidentified etiology, especially after a second attack of idiopathic AP, genetic counseling and/or testing should be considered [3,4].

## 5. Assessment of Severity of Acute Pancreatitis

Determining the severity of acute pancreatitis is important for patient management, as well as for patient prognosis. It enables the appropriate therapeutic action to be taken at the adequate time, thus preventing the development of further inflammatory changes of the pancreatic parenchyma and peri-pancreatic tissues, further complications, organ failure, and, eventually, the death of the patient [2]. To establish the severity of AP, clinical evaluation (including cardiovascular, respiratory, and renal systems), chest X-ray, calculation of body mass index and an APACHE II score (or other severity-of-the-disease rating systems), as well as research for any organ failure, should be immediately performed, while to predict complications in AP, reliable and accessible prognostic features are used: clinical impression of severity, obesity, serum C-reactive protein (CRP) levels > S150 mg/L, and other laboratory assays and a Glasgow score equal to or more than 3, an APACHE II > 8, during the first 24 h, or persisting organ failure after 48 h of hospitalization [51]. Additionally, a few scoring systems concerning clinical and laboratory criteria have been devised: APACHE, Bedside Index of Severity in Acute Pancreatitis (BISAP), and Ranson’s Glasgow. Determining the value of the elevation of lipase and amylase does not predict the severity of AP in adults. However, lipase levels might be referred to for the disease severity among children. A study by Basnayake and Ratnam [11] showed that a serum lipase activity more than seven times greater than the upper limit of normal during first 24 h can be a simple clinical predictor of severe AP in children.

On admission, to predict the outcome of AP, it is recommended to follow a 3-dimension approach concerning host risk factors (e.g., age, co-morbidity, and body mass index), clinical risk stratification (e.g., persistent SIRS), and the monitoring of the response to initial therapy (e.g., persistent SIRS, blood urea nitrogen, and creatinine) [4]. According to many studies, the independent risk factors for severe AP, as well as local complications or death, are BMI, overweight, and/or obesity. It has been confirmed that changes in intra-abdominal pressure (IAP) and BMI were significantly associated with the severity of AP. Furthermore, it has been suggested that the new approach using BMI and IAP has greater sensitivity and specificity than severity-of-the-disease rating systems, such as APACHE-II, BISAP, CTSI, and Ranson’s score [3,71,72,73].

### 5.1. Serum C-Reactive Protein and Other Laboratory Assays

Helpful predictors of the severity of AP, listed according to the time when they present the highest clinical value, are serum procalcitonin and urinary TAP and trypsinogen-2 on admission, serum interleukins-6 and -8 and polymorphonuclear elastase at 24 h, and CRP at 48 h [15]. An available and inexpensive way to predict the severity of acute pancreatitis is to measure the level of the aforementioned CRP. CRP gradually increases with the severity of acute pancreatitis and generally peaks 36–72 h after the onset of symptoms; therefore, its measurement is not applicable to the determination of disease severity on admission [2,74,75]. According to Mayer et al. [74], based on a prospective multicenter study, serum amyloid A (SAA), which is an early and sensitive marker of the extent of tissue damage and inflammation, is a better early marker of severity in acute pancreatitis on admission and during the first 24 h from onset of symptoms than the CRP measurement. The CRP measurement should be performed on admission and daily for the first 72 h after admission. If, at the beginning of the patient’s stay in the hospital or within the first 72 h, the CRP level is 14 286 nmol/L (150 mg/dL) or more, this suggests acute pancreatitis and is also associated with a worse clinical course [2,74].

However, if similar values are present within 48 h from admission, it is helpful in the distinction between severe and mild disease [2]. The occurrence of necrosis has been associated with CRP levels greater than 17,143 nmol/L (180 mg/dL) in the first 72 h after disease onset [2]. Additionally, it was discovered that daily observation of serum procalcitonin provides a non-invasive detection of infected necrosis [15]. However, the study from 2019 by Farkas et al. [76] suggested that neither CRP nor WBC can predict mortality or severe disease on the day of admission, even if tests are restricted to patients who present within less than 24 h from the onset of the pain.

Other laboratory outcomes used to characterize an episode of severe acute pancreatitis are blood urea nitrogen (BUN) >  20 mg/dL (> 7.14 mmol/L) or rising BUN, hematocrit (HCT) >  44% or rising HCT, procalcitonin, and lactate dehydrogenase (LDH) [3]. Their significance in severe AP is characterized in the section about the management of AP.

### 5.2. Severity-of-Disease Rating Systems

There are plenty of rating systems, including, among others, Ranson’s criteria (1974), the Glasgow-Imrie score (1978), the Acute Physiology and Chronic Health Evaluation II (APACHE II), the Simplified Acute Physiology Score (SAPS II) (1984), the Sequential Organ Failure Assessment (SOFA), the CT severity index (CTSI), the Bedside Index of Severity in Acute Pancreatitis (BISAP) score (2008), and Japanese Severity. Most scores are based on patient demographics, clinical features, laboratory parameters, or imaging modalities, and are assessed on admission or within 48 h. The majority of the aforementioned scoring systems include the following predictors: age, organ failure or immunocompromise, a previous history of chronic disease, temperature, blood pressure, pulse rate, respiratory rate, body mass index, consciousness level, presence of peritonitis, presence of acute renal failure, white blood cell count, blood hematocrit, blood platelet count, blood glucose, blood urea nitrogen, serum creatinine, serum aspartate transaminase, serum lactate dehydrogenase, serum calcium, serum electrolytes, serum bilirubin, plasma albumin, oxygen saturation, pH, base deficit, and multiple imaging modalities, mainly CT [3,71].

There is no “gold standard” prognostic score for predicting severe AP. Presumably, the Bedside Index of Severity of Acute Pancreatitis (BISAP) score is one of the most precise and applicable in everyday clinical practice because of the simplicity and the capability to predict severity, death, and organ failure. BISAP includes five variables: blood urea nitrogen levels > 25 mg/dL, impaired mental status or Glasgow Coma Scale GCS < 15, SIRS, age > 60 years, and pleural effusion on imaging. [77].

Ranson’s criteria are one of the earliest scoring systems to assess the severity of AP. The criteria are named after Dr. John Ranson, a surgeon and leading figure on the pancreas during the 20th century. The original Ranson’s criteria use 11 parameters to assess the severity of AP, namely age, white blood cell count, blood glucose, serum aspartate transaminase, serum lactate dehydrogenase, serum calcium, fall in hematocrit, arterial oxygen, blood urea nitrogen, base deficit, and sequestration of fluids. It should be mentioned that there is also a modified version of Ranson’s criteria. The original criteria with 11 parameters are used to score alcoholic pancreatitis, while the modified criteria have 10 parameters, which are used to score gallbladder pancreatitis [78]

The Ranson’s score has poor accuracy in classifying the severity of AP; it also requires a full 48 h to be completed; so, it cannot be used in a potentially valuable early therapeutic window, whereas the BISAP score contains data that can be evaluated on admission and are accurate in predicting a patient’s outcome within 24 h. Ranson’s score requires lots of variables that raise the cost of complete diagnosis and management, while the BISAP score has less variables, and they are cost-effective and can be performed in an emergency setting [77].

Recently, the harmless acute pancreatitis score (HAPS) has been introduced to identify AP with a non-severe course. HAPS is an accessible and useful scoring algorithm that efficiently and rapidly identifies patients who will develop a non-severe course of AP. Assessment can be completed within one hour from admission. For comparison, the Ranson’s score, although more accurate, takes 48 h to complete [79,80,81].

Another rating system that can be evaluated on admission and daily for the first 72 h after admission is the calculation of the APACHE II score [2]; this is the most frequently used severity-of-disease rating system in ICUs worldwide. Within the first 24 h after admission, several physiological variables are used to calculate an overall score from 0 to 71. As the score increases, the probability of more severe disease and hospital mortality is higher. For instance, APACHE II values 35–100 correspond to a mortality of 85% [82]. If the APACHE II score is 8 or higher at the beginning of admission or during the first 72 h, this suggests severe acute pancreatitis and a worse clinical course. It has also been noted that in the aforementioned time range, there is a correlation between higher APACHE II scores and higher patient mortality. Mortality with APACHE II scores less than 8 approximates <4%, while with scores greater than or equal to 8 it reaches 11–18%. The dynamics of the changes in the APACHE II score are important in differentiating the severity of acute pancreatitis. Namely, an increase in the APACHE II score in the first 48 h strongly suggests the development of severe acute pancreatitis, while a decrease within the first 48 h is a strong predictor of mild acute pancreatitis. Despite the usefulness of the APACHE II scale, it has its drawbacks. One of them is a limitation in the possibility of identifying the severity of AP in patients, such as when distinguishing between interstitial and necrotizing acute pancreatitis [83,84,85]. Kumar and Griwan [86] presented a prospective comparison of several scoring systems and determined their usefulness in predicting the severity of acute pancreatitis according to the Atlanta 2012 definitions, which classify acute pancreatitis on the basis of easily detectable clinical and radiological criteria. Researchers compared scoring systems that use biochemical and clinical data, such as APACHE II, BISAP, and Ranson’s score, and an imaging-based indicator called the modified Computed Tomography Severity Index (CTSI).

Assessment of the severity of acute pancreatitis can be conducted by the Balthazar CTSI scoring [87] and Mortele Modified CTSI scoring [88]. Balthazar et al. [87], in 1990, created CTSI by combining the original grading system with the presence and extent of pancreatic necrosis. The score obtained with the index did not significantly correlate with the subsequent development of organ failure, extra pancreatic parenchymal complications, or peripancreatic vascular complications [87]. Due to this limitation, in 2004 Mortele et al. [88] created a modified CT scoring system to determine whether the CTSI could be used to predict the clinical outcome more accurately. The Mortele modified CTSI was easier to calculate and was found to correlate more closely with patient outcome measures, such as the length of the hospital stay, the need for surgery/intervention, and the occurrences of infection, organ failure, and death, than the currently accepted Balthazar CTSI, with similar inter-observer variability [88].

The Balthazar CTSI is calculated by adding the points related to the CT image and the points referring to the presence and extent of the necrosis. Subsequently, the total score is categorized as mild, moderate, or severe pancreatitis (Table 4). The Balthazar CTSI scoring differs from the modified CTSI scoring in the method of scoring a CT image and in assessing the extent of necrosis [48].

The modified CTSI is calculated by summing the evaluated parameters, and the total score is then categorized, similarly to that of the Balthazar CTSI, as mild, moderate, or severe pancreatitis [48]. Additionally, 2 points are added to the sum of points if the extrapancreatic findings are present (Table 5).

According to the revised Atlanta classification [1], the severity is classified into three categories. As mentioned above, mild pancreatitis is diagnosed if organ failure and local or systemic complications are absent. Moderate pancreatitis means the presence of transient organ failure of less than 48 h and/or the presence of local complications. Finally, severe pancreatitis occurs in the case of persistent organ failure for more than 48 h.

The comparative evaluation performed by Bollen et al. [89] comparing CTSI and modified CTSI and comparing both CTSI indices with the APACHE II scale concluded that the modified CTSI is better than CTSI in assessing the severity of acute pancreatitis, while the CTSI is better than the APACHE II scale in assessing the severity of acute pancreatitis.

Kumar and Griwan [86] stated from their research that the APACHE II scale is slightly more useful than the Ranson’s score and BISAP in predicting the severity of acute pancreatitis. Research carried out by Raghuwanshi et al. [48] concerned another two CT severity indices, which are used to evaluate the severity of AP: Balthazar’s CTSI and the modified Mortele CTSI. Both CT severity indices are based on inflammation of the peri-pancreatic fat, which is a reflection of intrinsic pancreatic abnormalities associated with hazy, streaky densities and fluid collection (phlegmon), as well as the presence and extent of necrosis [48].

According to the results of the study, the modified Mortele CTSI is calculated more easily and correlates more accurately with patient outcome parameters, such as duration of hospitalization, requirement for surgery/intervention, presence of infection, organ failure, and death, than the Balthazar CTSI. However, the revised Atlanta classification is found to be more precise than the modified Mortele CTSI and Balthazar CTSI in evaluating the mortality and organ failure [48,90].

The disadvantages of known severity-of-the-disease rating systems are that most of the earlier prognostic scores need at least 24 h to predict severity, and several parameters are not easily available on admission. Therefore, early prediction of AP severity is still needed. The early achievable severity index (EASY) is a multinational, multicenter, prospective, and observational study. The EASY prediction score is a practical tool for identifying patients at high risk for severe AP within hours of hospital admission. The aim is to predict whether a patient will develop severe or non-severe AP on the basis of the data obtained at the time of hospital admission. The features that can be evaluated are age, gender, BMI, alcohol consumption, smoking habits, duration of abdominal pain, blood pressure and pulse, body temperature, respiratory rate, abdominal point tenderness, abdominal muscular reflex, and the results of a blood test (amylase, aspartate aminotransferase, glucose, urea nitrogen, creatinine, CRP, sodium, potassium, calcium, and WBC count). The top six most influential features regarding all cohorts were creatinine, glucose, respiratory rate, urea nitrogen, white blood cell count, and gender. The web application is available for clinicians and contributes to the improvement of the model [91].

Most rating systems used to predict the severity of AP have focused on death as an outcome. Over the last decades, the mortality of AP has been declining; therefore, it should be considered whether death should remain as the main outcome to predict AP. According to the IAP/APA evidence-based guidelines for the management of acute pancreatitis, systemic inflammatory response syndrome (SIRS) is advised to predict severe AP at admission and persistent SIRS at 48 h. To diagnose SIRS, the presence of two or more of the following four criteria is needed: temperature < 36 °C or >38 °C, heart rate >90/min, respiratory rate >20/min, and white blood cells <4 × 109/L (<4 K/mm^3^), >12 × 109 (>12 K/mm^3^) or 10% bands. Persistent (>48 h) SIRS is associated with multi-organ failure and mortality in AP, whereas persistent (>48 h) organ failure is the principal determinant of mortality in AP. Systemic inflammatory response syndrome (SIRS) is advised to predict severe acute pancreatitis at admission and persistent SIRS at 48 h. There are many different scoring systems used to predict the outcome of AP, such as BISAP or APACHE II, as well as serum markers, such as CRP, hematocrit, procalcitonin, or BUN. The usefulness and effectiveness of (persistent) SIRS is comparable to the aforementioned scoring systems and markers for predicting severe AP [3,4].

### 5.3. Assessment of Organ Failure

A further way to assess the severity of AP is the evaluation of organ failure. If a patient, despite adequate intravenous fluid resuscitation, presents signs of persistent organ failure for more than 48 h, a diagnosis of acute AP should be made [2].

Banks et al. [1] presented in their research, which is a revision of the Atlanta Classification, the modified Multiple Organ Dysfunction Score [92], which was constructed using the physiologic indicators of failure in six organ systems. The organ failure-based criteria, taken partly from the aforementioned scoring system, are used in order to predict the severity in AP.

As assumed by Johnson and Abu-Hilal [93], the duration of organ failure during the first week of suspected severe AP is firmly related to the risk of local complications and patient death. They examined the mortality and morbidity associated with transient organ failure (resolving in <48 h) and persistent organ failure (lasting >48 h) in a group of patients with early (within the first week) AP-related organ failure. The results were as follows: the group of patients with transient organ failure had lower mortality rates (1%) and fewer of them experienced local complications of AP (29%) compared to the group of patients with persistent organ failure, where the above-mentioned parameters were 35 and 77%, respectively, whereas a good prognosis can be suggested in the case of resolution of organ failure within 48 h [93].

Another study examining organ failure in the course of AP is research carried out by Mofidi et al. [94], which evaluated the importance of the early systemic inflammatory response syndrome (SIRS) in the development of the multiorgan dysfunction syndrome (MODS) and death resulting from AP. Patients with AP who experienced SIRS with a duration of more than 48 h had a considerably higher value of multiorgan dysfunction, as determined by the Marschall Score, and had a higher mortality rate than patients with transient SIRS lasting less than 48 h. Based on the results, they concluded that SIRS is related to MODS and death in AP and is an early indicator of the likely severity of acute pancreatitis [94].

## 6. Management of Acute Pancreatitis

### 6.1. Supportive Care

The basic treatment of patients with mild AP is to provide supportive care, which includes resuscitation with isotonic intravenous fluids, pain control, continuous monitoring of vital signs, and evaluation of organ function [3,51].

Due to the harmful effects of fluid overload, fluid resuscitation should depend on frequent reassessment of hemodynamic status. The parameters that are recommended to be controlled are the laboratory indicators of volemia and adequate tissue perfusion: hematocrit, lactate, creatinine, and blood urea nitrogen [3]. As for the fluid volume required to prevent pancreatic necrosis or improve results, it is determined on the basis of the patient’s age, weight, and pre-existing renal and/or cardiac disorders [95]. High-quality studies investigating the effectiveness of aggressive fluid resuscitation in AP patients have not been conducted. However, this procedure promotes pancreatic microcirculation, owing to more comprehensive fluid administration, and thus prevents necrosis of the pancreas, providing a decrease in mortality among the patients during the last decade [2,3]. In addition, more cases of death and pancreatic necrosis have been reported in patients with hemoconcentration [2]. Moreover, it is worth mentioning that fluid resuscitation should be started before imaging.

Appropriate immediate fluid resuscitation is relevant in preventing systemic complications. Some evidence indicates the importance of providing AP patients with adequate early oxygen supplementation and fluid resuscitation until the risk of organ failure has been excluded, as these treatments are associated with the resolution of organ failure [34,51], while the latter correlates with low mortality [93]. In contrast, inadequate supportive care leads to organ failure and the subsequent local complications and eventual death of the patient. Johnson and Abu-Hilal [93] concluded that the risk of local complications or death is affected by the duration of organ failure during the first week of severe AP. The resolution of organ failure within 48 h is a good prognosis, while persistent organ failure is a marker of the following local complications and patient death.

The continuous measurement of oxygen saturation and the administration of supplemental oxygen are recommended to maintain arterial saturation at a level greater than 95%. Intravenous fluids, i.e., crystalloid or colloid, are given to maintain a urine output of 0.5 mL/kg body weight. UK guidelines from 2005 mention the use of crystalloids or colloids as needed [51], while the 2019 WSES guidelines suggest the isotonic crystalloids to be the preferred fluid [3]. Fluid resuscitation should be monitored by frequent central venous pressure measurements. It is also recommended to treat each patient intensively until the severity of the disease is established [51]. After carrying out an observational study, Alphonso Brown et al. [34] concluded that fluid resuscitation does not prevent pancreatic necrosis; however, insufficient fluid resuscitation, determined by an increase in hematocrit at 24 h, contributed to the development of necrotizing pancreatitis in all the studied patients.

Another study, a randomized controlled trial (RCT) performed by Wu et al. [3], compared the usage of Ringer’s lactate and normal saline in standard and goal-directed fluid resuscitation among the AP patients. The study presents the predominance of Ringer’s lactate over normal saline. Fluid resuscitation with Ringer’s lactate provides an 84% reduction in the incidence of SIRS in patients and causes a considerable decrease in the CRP level (from 104 mg/dL to 54 mg/dL) [46]. The changes caused by Ringer’s lactate may be related to the anti-inflammatory effect and a better adjustment of the potassium level [3].

The clinical priority and the essential part of the supportive management of patients with acute pancreatitis is pain relief—the fundamental symptom of AP. It is recommended to use a multimodal analgesic regimen, i.e., nonsteroidal anti-inflammatories, narcotics, and acetaminophen, in the first 24 h of hospitalization, if there are no contraindications, in order to improve the patient’s quality of life [2,3,96,97]. A study by Meng et al. [97] suggested that there are no certain data on the preference for a specific analgesic and the best method of administration in the course of AP. Therefore, it is recommended to follow the latest acute pain management guidelines in the perioperative setting [98,99]. However, there are some pain control strategies in the course of AP. In non-intubated patients, administration of dilaudid over morphine or fentanyl is preferable. Following the multimodal regimen, intravenous analgesia is an alternative or agonist to epidural analgesia [3], whereas epidural analgesia can be used in patients with severe and acute critical pancreatitis who need high doses of opioids for a prolonged period [98,99]. In addition, in the case of acute kidney injury, non-steroidal anti-inflammatory drugs (NSAID) should not be administered. Moreover, in each strategy described, patient-controlled analgesia (PCA) should be provided [3]. According to Basurto Ona et al. [96], in the AP-associated pain treatment a suitable selection may be opioids. The research shows that opioids may reduce the need for supplementary analgesia in comparison with other analgesics. Additionally, there is no evidence for a difference in the risk of complications or adverse effects in the course of AP as a result of opioids and other analgesia options administration [96].

Due to the initial treatment, consisting of aggressive fluid resuscitation, pain control, and assessment of organ function, continuous vital signs monitoring is essential. Vital signs monitoring is necessary in a high-dependency care unit if the patient develops organ dysfunction, while in the case of persistent organ dysfunction or development of organ failure, despite sufficient fluid administration, admission to the ICU is indicated [3]. Clinicians distinguish three criteria, in the case of which transfer to the monitored unit should be considered. The first criterion concerns the patients with acute pancreatitis diagnosed on the basis of CRP greater than 14,286 nmol/L (150 mg/L), an APACHE II score greater than 8, or organ dysfunction for more than 48 h despite sufficient fluid administration. Another criterion is characterized by the presence of symptoms indicative of current or progressing organ dysfunction, as defined by the following information presented in Table 6 [2]:

The third criterion includes the patients presenting severe hemoconcentration, i.e., hematocrit [HCT] > 0.500 and hemoglobin [Hb] > 160, and thus requiring aggressive, continuous fluid resuscitation. Additionally, there is one more recommendation that takes into account the above-mentioned criteria. Namely, patients with one or more of the aforementioned criteria, with a BMI greater than 30 (in Asian populations greater than 25), should be monitored thoroughly, with a lower threshold for transfer to a monitored care unit. This is due to reports which state that the obese patient population represents a worse course of the disease. Severe acute pancreatitis is associated with an increased risk of death, while symptoms of organ failure are seen in deceased patients. Patients with organ failure lasting more than 48 h during the first week of illness are at the greatest risk of death [100]. Due to the above-mentioned aspect, all patients with suspected severe acute pancreatitis should be carefully monitored, preferably in a high-dependency unit, whereas in patients with organ dysfunction or organ failure, supportive care should be ensured in ICU [51]. The work of Nathens et al. [101], concerning the management of critically ill patients with severe acute pancreatitis, which is a systematic review of 26 observational studies, showed that being cared for by an intensivist or the use of an intensivist consultation model in a closed ICU results in a shorter stay in the ICU and lower mortality in critically ill patients compared with similar patients who did not receive suchlike specialized intensivist care [101].

Acute pancreatitis can be complicated by organ failure. Therefore, guidelines for the management of patients with AP [3] include recommendations concerning mechanical ventilation as a treatment of respiratory failure, defined as the presence of Pao2/FiO2 ≤ 300 and respiratory rate > 20 breaths per min [1,2]. There are no specific issues with treating respiratory failure in AP patients. In the case that oxygen supplementation through high flow nasal oxygen or continuous positive airway pressure (CPAP) becomes inefficient in improving tachypnoea and dyspnea, mechanical ventilation must be introduced. Non-invasive, as well as invasive, techniques can be used. However, if clearing bronchial secretions becomes ineffective and/or the patient is tiring or predicted to tire, invasive ventilation is obligatory. Additionally, the use of invasive ventilation in patients is an indication for the use of lung-protective strategies [3]. Oxygen supplementation can become ineffective in treating respiratory failure despite the use of high-flow nasal oxygen or CPAP. Hypoxia only explains in part the different levels of tachypnoea and dyspnea. Despite appropriate arterial oxygenation, pleural effusion, possible intra-abdominal hypertension, or pain can lead to these symptoms. Furthermore, after fluid resuscitation, pulmonary edema may occur, which is accelerated by increased systemic permeability [28,102].

Another important element of supportive care is the control of intra-abdominal pressure. The aggravation of intra-abdominal hypertension and intestinal failure result from increased systemic permeability caused by systemic inflammation and therapeutic measures such as vasoactive drugs or fluid resuscitation, while excessive sedation can additionally exacerbate dysfunction of the intestines with the consecutive enhancement of intra-abdominal pressure. Prior to surgical abdominal decompression, if all other non-operative treatments (e.g., percutaneous drainage of intraperitoneal fluid) are insufficient, deep sedation and paralysis are considered to be mandatory in the limitation of intra-abdominal hypertension. It is suggested to restrict vasoactive drugs, fluids, and sedation in order to accomplish resuscitative goals at lower normal limits. Furthermore, limitation of the medicaments usually used at ICU when side effects overcome advantages is essential [3,103].

### 6.2. Improvement of Laboratory Parameters

The severe AP is defined as AP with persistent organ failure (lasting more than 48 h), with a mortality rate of 20–50% [1]. Early assessment of disease severity is crucial for the determination of the therapeutic strategy because effective treatment could significantly decrease the mortality of patients with severe AP. There are invasive or non-invasive methods used for evaluating the severity of AP, including scoring systems, radiological imaging modalities, and biochemical parameters. During management of the patient, laboratory parameters, such as albumin, glucose, HTC, TG, CRP and procalcitonin, creatinine, BUN, and the estimated glomerular filtration rate (eGFR), should be constantly monitored and corrected if possible [3,104].

Hypoproteinemia has been observed in AP patients. An abnormally low-level of albumin may be regarded as an essential starter in the pathogenesis of AP. The study by Li et al. [105] showed that low serum albumin on admission is independently associated with persistent organ failure in severe AP and suggested that albumin is a valuable tool for a rapid assessment of persistent organ failure in patients with AP.

High glucose level is one of the parameters used in Ranson’s criteria. This severity-of-the-disease rating system, as well as the other systems, is used to predict the mortality of AP, which is associated with the severity of AP. Glycemia is taken into account at admission. According to the original Ranson’s scoring, blood glucose above 200 mg/dL is significant, while according to the modified Ranson’s scoring it is above 220 mg/dL [3,78,81,86,106].

Serum triglyceride and calcium levels should be measured in the case of the absence of gallstones or a significant history of alcohol use. Serum triglyceride levels over 11.3 mmol/L (1000 mg/dL) indicate it as the etiology. Thus, it is the basis for the introduction of the appropriate management of hypertriglyceridemia-induced AP, namely lipid-lowering therapy [3,107].

Urea > 20 mg/dl can be used as independent predictor of mortality, while hematocrit > 44% can be regarded as an independent risk factor of pancreatic necrosis. Testing procalcitonin levels may be used to predict infected necrosis among the patients with confirmed pancreatic necrosis. A procalcitonin value of 3.8 ng/mL or higher within 96 h after the onset of symptoms indicated a pancreatic necrosis with a sensitivity and specificity of 93% and 79%. Procalcitonin is regarded as the most sensitive laboratory test for detection of pancreatic infection. Low serum values seem to be strong negative predictors of infected necrosis [108,109,110,111,112]. The serum level of LDH on admission can be used to predict severe AP, death, and admission to an intensive care unit (ICU), but it should be measured together with other laboratory parameters [106].

Persistent organ failure is associated with, among other factors, kidney insufficiency. In this state, eGFR is decreasing and creatinine tends to increase because of the renal injury and, additionally, pancreatic necrosis [113]. In the study by Lipinki, Rydzewki, and Rydzewska [114], both serum creatinine and eGFR measured on admission and 48 h later were significantly associated with the presence of pancreatic necrosis. Furthermore, the serum creatinine level and eGFR measured on the 1st day proved to be a good predictor of a fatal outcome. The presence of pancreatic necrosis and mortality were significantly higher among the patients with an elevated serum creatinine level and low eGFR values. They suggested that the serum creatinine level and eGFR can be useful indicators of mortality and pancreatic necrosis on admission.

In conclusion, low albumin, high glucose, high HCT, high triglycerides, and low eGFR ought to be constantly monitored and corrected if possible.

### 6.3. Nutrition

It is assumed that the fundamental pathogenesis of acute pancreatitis is premature, i.e., still within the pancreatic ducts, with the activation of the pancreatic proteolytic enzymes causing the process of autodigestion of the pancreas. According to previous practices, the inhibition of intestinal function, in other words bowel rest, was expected to reduce the inflammatory process in the pancreas caused by the autodigestion process [115]. However, recently performed RCTs show that early oral/enteral feeding in AP patients does not cause adverse effects and might even lead to significant reductions in pain, food intolerance, and opioid use [2].

The World Association guidelines and the Santorini consensus assume that enteral nutrition in AP is safe [3]; however, enteral nutrition in mild pancreatitis does not determine improvement, and these patients do not need to have any dietary restrictions. In the course of AP, artificial feeding can be used to provide long-term nutritional support and to prevent complications. It has been observed that nausea inhibits oral food consumption among patients with severe AP [51]. The acute inflammation is related to a disruption in the functioning of the intestinal mucosal barrier; thus, it is recommended to introduce enteral nutrition in order to prevent intestinal failure and infectious complications. This is because enteral nutrition preserves the intestinal mucosa barrier and prevents disruption and bacterial displacement that could potentially colonize pancreatic necrosis. It also restricts the stimulus to the inflammatory response, particularly among the patients managed with enteral nutrition in whom occurred the reduction in oxidant stress and systemic exposure to endotoxin [3,51,116].

The research carried out by Windsor et al. [18] compared enteral feeding with parenteral nutrition and results in the conclusion that enteral feeding reduces the acute phase response and alleviates the severity of acute pancreatitis and clinical outcome, though with the absence of changes in pancreatic injury on the CT scan. Given these facts, enteral nutrition appears to be clinically beneficial and safer than parenteral nutrition and causes fewer septic complications [18,51] Concerning the method of administration, continuous infusion is more frequently used in comparison with cyclic or bolus administration in the majority of institutions [3].

On admission, the standard diet is recommended for patients with mild AP. If, due to symptoms occurring in the patient, i.e., nausea, vomiting, abdominal pain or ileus, the patient does not tolerate the oral diet, the patient is allowed to self-adjust the diet to the current state, to withhold from oral food and fluids or to follow a regular diet [2]. However, the study of Eckerwall et al. [117] showed that compared to withholding oral food and fluids, oral nutrition on admission markedly reduced the time of hospitalization from 6 to 4 days in patients with mild AP. Only when feeding is started within the first 48 h after admission can the main benefits from early feeding be observed in patients. Therefore, according to the recommendation, oral feeding should be commenced on admission if tolerated or within the first 24 h [118].

During recovery from mild pancreatitis, patients are initially fasted and, when they begin to tolerate oral nutrition, they receive oral refeeding with a clear liquid diet (CLD) in order to avert unfavorable gastrointestinal events. The diet is gradually changed to soft solids. Discharge from hospital depends on whether the dietary change is effective, and the patient tolerates solid food. The research of Jacobson et al. [119] from 2007 and the study by Sathiaraj et al. [120] from 2008 showed that after mild acute pancreatitis it is safe to initiate oral nutrition with a low-fat solid diet (LFSD) instead of a CLD, and it also provides more calories. However, the results of the studies differ in terms of the shortening of hospitalization time; Sathiaraj et al. [120] showed that the above-mentioned treatment strategy can reduce the length of hospitalization, while Jacobson et al. [119] proved that there is no such connection. Nevertheless, the dominance of a low-fat diet over a regular diet has not been proven [2].

Enteral feeding in predicted mild pancreatitis can be resumed once inflammatory markers are improving and abdominal pain is decreasing. There is no need to postpone introducing enteral feeding until the laboratory abnormalities or pain are completely resolved. It has been shown that immediate oral refeeding with a normal diet is safe in predicted mild pancreatitis; it also accelerates recovery and leads to a shorter hospital stay without adverse gastrointestinal events. Additionally, starting with a liquid or soft diet is unnecessary; feeding can be started with a full solid diet in the case of predicted mild pancreatitis [4,117,121].

In the case of severe acute pancreatitis, enteral nutrition should be introduced within 48 h of admission [2]. The work of Kalfarentzos et al. [122] suggested the predominance of using early enteral nutrition in patients with severe acute pancreatitis over parenteral nutrition due to fewer complications, the lower risk of developing septic complications, and the lower cost of the procedure. According to the recommendations, the use of enteral nutrition is preferable to parenteral nutrition in patients with AP and severe AP. Furthermore, numerous RCTs establish that, compared to parenteral nutrition, enteral nutrition reduces mortality, local septic complications and other complications, systemic infection, surgical interventions, and multiorgan failure. In addition, it has been suggested that the use of enteral nutrition instead of parenteral nutrition shortens the length of hospitalization [10,24].

Results similar to the above-mentioned findings are achieved when enteral nutrition is administered within the first 48 h after admission [123]. Total parenteral nutrition should be avoided in the course of acute pancreatitis and severe acute pancreatitis. However, in the case of incomplete tolerance of the enteral feeding route, it is recommended to consider the use of partial parenteral nutrition in order to ensure the caloric and protein requirements [3]. In the study of Gupta et al. [124], comparing the use of early enteral nutrition with parenteral nutrition, no significant clinical benefits in recovery time have been revealed. Moreover, enteral nutrition may be restricted by ileus. If an ileus lasts for more than 5 days, the introduction of parenteral nutrition is mandatory [51].

The safety of both gastric and jejunal nutrition has been demonstrated; however, there is no dominance of a nasojejunal tube over a nasogastric tube. For this reason, the administration of feeding should not be delayed because of the insertion of a nasojejunal feeding tube [3].

Based on prospective studies, Petrov et al. [125] indicated no change in mortality and food intolerance during enteral nutrition through a nasogastric tube, as well as through a nasojejunal tube, whereas Chang et al. [126], after analyzing several RCTs, suggested that there are no considerable differences in the aggravation of pain, diarrhea, tracheal aspiration, energy balance achievement, and mortality between nasogastric tube-fed patients and nasojejunal tube-fed patients. In the case of necrotizing pancreatitis, the administration of early nasoenteric tube nutrition within 24 h after randomization, compared to the oral diet commenced 72 h after admission, provided no reduction in the incidence of infection or death [3]. In most studies, enteral feeding through a nasojejunal tube is used during the management of acute pancreatitis. However, in up to 80% of patients, nasogastric nutrition seems to be effective. Due to the risk of the aspiration of refluxed feed, nutrition through the nasogastric tube of incompletely conscious patients should be administered with caution [51].

Various formulations are used in the course of pancreatitis, but there is no evidence of the relative advantages of standard, elemental, partially digested, or immune-enhanced formulations [51] According to the research carried out by Petrov et al. [127], as well as Sun et al. [99], there is insufficient evidence to support the clinical benefits of using immune-enhanced, semi-elemental, and probiotic enteral feeds in the management of severe AP [99,127].

### 6.4. Pharmacological and Antimicrobial Treatment

No specific drug therapy has been proven to be effective in the treatment of acute pancreatitis [3,51]. Broad randomized studies have shown no clinically significant benefits in the management with anti-inflammatory agents, e.g., lexipafant and antiproteases, e.g., gabexate, as well as antisecretory agents, e.g., octreotide [128,129,130]. Furthermore, in patients with mild or severe acute pancreatitis, prophylactic antibiotic treatment is not recommended [2,3]. However, in the case of infected severe acute pancreatitis, the most serious local complication of acute pancreatitis, the use of antibiotics is always advised [3,51].

Gram-negative bacteria dominate in pancreatic infection; however, Gram-positive bacteria, anaerobes, and sometimes fungi are also present [131]. The most common fungi that can be found in the infected pancreas are *Candida albicans*, *Candida tropicalis*, and *Candida krusei* [3]. The use of antibiotics with proven penetration into pancreatic necrosis is recommended in the case of infected necrosis. The spectrum of empirical antibiotic therapy should include aerobic and anaerobic and Gram-positive and Gram-negative microorganisms [3].

Intravenously administered aminoglycosides do not penetrate the pancreas sufficiently to achieve the minimum inhibitory concentration of the bacteria found in secondary pancreatic infections [132] Acylureidopenicillins and third-generation cephalosporins show moderate penetration into pancreatic tissue; however, they achieve the MIC for most of the Gram-negative bacteria found in pancreatic infections. Only piperacillin/tazobactam, belonging to this group of antibiotics, is effective against Gram-positive anaerobes [133]. Quinolones (ciprofloxacin and moxifloxacin), carbapenems, and metronidazole are antibacterial drugs with good tissue penetration into the pancreas and effect against anaerobic bacteria [3,134]. However, quinolones should only be used in patients allergic to beta-lactam antibiotics, due to the high rate of resistance among bacteria worldwide. In addition, due to carbapenem-resistant *Klebsiella pneumoniae*, carbapenems should only be used in critically ill patients [3].

A serious complication of acute pancreatitis is a fungal infection, which is associated with an increase in morbidity and mortality. Nevertheless, due to the insufficient number of studies, the prevention of fungal infections is not recommended [135].

As mentioned earlier, given that pain is the primary symptom of acute pancreatitis, all patients with AP must receive some form of analgesia. However, no specific analgesics are used. It is recommended to adhere to the latest acute pain management guidelines in the perioperative setting [3].

### 6.5. Surgical and Operative Treatment

The treatment of acute gallstone pancreatitis is based on ERCP. The procedure is not performed routinely. The indications for ERCP in the course of acute gallstone pancreatitis are acute gallstone pancreatitis and cholangitis and acute gallstone pancreatitis with common bile duct obstruction [3].

In the event of signs or a strong suspicion of infected necrotizing pancreatitis, along with the accompanying clinical deterioration, it is recommended to undertake an intervention, namely percutaneous or endoscopic drainage. Walled-off pancreatic necrosis (WOPN) is a mature, encapsulated collection of pancreatic and/or peripancreatic necrosis with an inflammatory wall, which occurs most often after 4 weeks after the onset of the disease. Intervention for necrotizing pancreatitis should be initiated when the necrosis has become walled-off, namely after approximately 4 weeks, as mentioned above. The indications for carrying out the above intervention after 4 weeks after the onset of the disease are a symptomatic or growing pseudocyst, disconnected duct syndrome, on-going organ failure without sign of infected necrosis, and on-going gastric outlet, biliary, or intestinal obstruction (due to a large walled off necrotic collection), whereas the indication for percutaneous or endoscopic drainage of pancreatic collections after 8 weeks after the onset of the disease is on-going pain and/or discomfort [3].

If percutaneous or endoscopic interventions do not improve the patient’s condition, then surgical strategies should be considered. The indications for surgical treatment are abdominal compartment syndrome, bowel ischemia or acute necrotizing cholecystitis during acute pancreatitis, bowel fistula extending into a peripancreatic collection, and acute on-going bleeding (when an endovascular approach is unsuccessful). The procedure is also performed as a continuum in a step-up approach after a percutaneous or endoscopic procedure with the same indications [3]. The Eastern Association for the Surgery of Trauma [136] has shown that later surgery improves patient survival. Thus, it is recommended to perform surgical intervention more than 4 weeks after the onset of the disease, to reduce the likelihood of death [3]. This is due to the fact that over time the necrosis becomes more separated from the vital tissue, which ensures less damage to the vital tissue during the procedure. This increases the probability of less bleeding and more effective necrosectomy during late surgery [3].

According to the International Association of Pancreatology (IAP) recommendations [137], mild AP is not an indication for pancreatic surgery, while in mild gallstone-associated AP it is recommended to perform cholecystectomy, preferably during the same hospitalization after the patient has returned to a relative stability. Surgical treatment of acute gallstone pancreatitis concerns cholecystectomy with operative cholangiography [4,137,138]. Surgical treatment is not used in most patients with acute pancreatitis, while many patients eventually undergo cholecystectomy [51]. According to the guidelines of the IAP, this procedure is performed to prevent the recurrence of gallstone-associated acute pancreatitis [137]. If ERCP and sphincterotomy are performed, the same admission cholecystectomy is recommended in order to avoid other biliary complications. If peripancreatic fluid collections develop in acute gallstone pancreatitis, cholecystectomy should be postponed until the fluid collection has subsided or has stabilized and acute inflammation has resolved [3].

Only if specific indications occur in patients with necrotizing pancreatitis is it recommended to perform early surgery within 14 days following the onset of the disease [51]. In patients with a disconnected pancreatic duct, and in selected cases with walled-off necrosis, a minimally invasive surgery—transgastric endoscopic necrosectomy—may be considered. However, percutaneous drainage is recommended as the first line of treatment in infected pancreatic necrosis. It allows the postponement of surgical treatment to a more appropriate time, and it can also lead to a complete resolution of infection in 25–60% of cases [3]. The use of an open abdomen in order to perform surgical decompression is effective in treating the abdominal compartment syndrome in patients with severe acute pancreatitis unresponsive to the conservative management of intra-abdominal hypertension or abdominal compartment syndrome. It is recommended to avoid the open abdomen procedure if other strategies can be used to treat or alleviate severe intra-abdominal hypertension in severe acute pancreatitis [3].

It is worth emphasizing that, in any case, the surgical strategies should be considered if endoscopic procedures fail to improve the patient’s condition [3].

## 7. Prevention of Acute Pancreatitis

It is crucial to identify the etiology of AP on admission in order to introduce the best and, in the case of some patients, the most specific therapy. The best intervention in biliary AP with cholangitis is early ERCP; in hypertriglyceridemia-induced AP it is the introduction of lipid-lowering therapy; in the case of obstruction-evoked AP the pancreatic stent placement pancreatic duct is performed, while in autoimmune pancreatitis the steroid therapy is implemented [107].

The study by Zádori et al. [107] has shown that 5% of the patients left the hospital after the first and second attacks of AP without any imaging at all, while 25% of patients had no diagnostic work-up for biliary AP, such as laboratory tests. The greatest insufficiency in etiology screening, amounting to 71–76%, concerned lipid-induced (triglyceride or cholesterol) pancreatitis. In the case of additional diagnostic work-up for all idiopathic AP after index admission, in 91% of the cases there was no search for biliary, anatomic, or cancer etiology by EUS or MRCP, for autoimmune AP in 98% of cases, for genetic AP in 99%, or for virus-induced AP after the first attack in 94%.

Unfortunately, the IAP/APA guidelines are insufficiently introduced into daily clinical practice. The etiology of AP remains unclear in almost a quarter of all cases, which can be caused by an insufficient diagnostic work-up or other unknown etiological factors. It is worth mentioning that the cause of about 40% of fatal AP cases is idiopathic, which emphasizes the significance of determining the etiology. Moreover, defining the etiology is crucial for index AP, as well as in preventing recurrent or chronic pancreatitis [107].

Therefore, in patients considered to have idiopathic AP, it is recommended to exclude biliary etiology by at least two US examinations in order to prevent recurrent pancreatitis. The subsequent step in diagnostics is EUS, performed after the acute phase of pancreatitis, in order to assess for occult microlithiasis, neoplasms, or chronic pancreatitis. If the EUS is negative, MRCP is recommended as the following step to identify rare morphologic abnormalities. In the case of unidentified etiology, especially after a second attack of idiopathic AP, genetic counseling and/or testing should be considered to diagnose hereditary pancreatitis or to understand the genetic risks of AP [3,4,107].

In the case of AIP, the diagnosis is made on the basis of clinical and radiological features, serological parameters, and pathological findings. According to the International Consensus Diagnostic Criteria for Autoimmune Pancreatitis (ICDC), type 1 AIP can be diagnosed by assessing a combination of five primary cardinal features: (1) imaging features of the pancreatic parenchyma, assessed by the use of CT or MRI, and the pancreatic duct, evaluated by ERCP or MRCP; (2) serum IgG4 level; (3) other organ involvement; (4) histopathology of the pancreas; and (5) response to steroid therapy, while the diagnosis of type 2 AIP is made by assessing a combination of four of the primary cardinal features from type 1, excluding serology [139,140].

Meta-analysis by Márta et al. [141] suggested a method to prevent post-ERCP pancreatitis, namely a combination of lactated Ringer’s and indomethacin. According to the results, aggressive hydration with indomethacin is more effective than single therapy and is also significantly more effective than all other interventions. They concluded that a one-hit-on-each-target therapeutic approach can be used in PEP prevention, with the usage of an easily accessible combination of aggressive hydration and indomethacin in the case of all average and high-risk patients without contraindication.

## 8. Conclusions

The paper presents the most important information on the diagnosis, evaluation, and treatment of acute pancreatitis. There are many laboratory tests and imaging diagnostic methods, but only some of them are useful in diagnosis; others are used to assess the severity of the disease process. The importance of non-invasive treatment, based on the supportive care and proper nutrition of the patient, introduced immediately, is worth attention. Drug treatment is non-specific, focused on the management of pain in accordance with the current guidelines. On the other hand, the basis of surgical treatment is minimally invasive percutaneous or endoscopic procedures, while open abdominal surgery is not recommended, with few exceptions. Overall, the review may be a useful summary of the information on acute pancreatitis for medical students and young clinicians.

## Figures and Tables

**Table 1:** Classification of acute pancreatitis based on the 2012 Atlanta Classification of Acute Pancreatitis. Explanation of crucial terms.

Severity of Acute Pancreatitis	Characteristics
Mild acute pancreatitis	The most frequent form No organ failure No local or systemic complications Usually resolves in the first week
Moderately severe acute pancreatitis	Transient organ failure resolving within 48 h Local or systemic complications without persistent organ failure Exacerbation of co-morbid disease
Severe acute pancreatitis	Persistent organ failure > 48 h
**Criterion of Acute Pancreatitis**	**Characteristics**
Organ failure and systemic complications of acute pancreatitis	Respiratory System: Pao2/FiO2 ≤ 300 Cardiovascular System: systolic blood pressure < 90 mm Hg (off inotropic support) not fluid responsive or pH < 7.3 Urinary System: serum creatinine ≥ 170 μmol/L
Local complications of acute pancreatitis	Acute peripancreatic fluid collections Pancreatic and peripancreatic necrosis (sterile or infected) Pancreatic pseudocyst Walled-off pancreatic necrosis (sterile or infected)

**Table 2:** Crucial information concerning indicator enzymes used in the diagnosis of acute pancreatitis, based on information published in the studies of Rompianesi et al., Matull et al., and Chase et al., as well as in other publications.

Assay	Serum Lipase	Serum Amylase
**Origin of the enzyme**	Pancreas [14]	Pancreas, salivary glands, small intestine, ovaries, adipose tissue, skeletal muscle [14]
**The normal range of the enzyme**	5-208 U/L [18]	30-110 U/L [18]
**The dynamics of enzyme level**	- Rise within 4–8 h; - Peak at 24 h; - Decrease to normal or near-normal levels over the next 8–14 days [22].	- Rise within 6–24 h; - Peak at 48 h; - Decrease to normal or near-normal levels over the next 5–7 days [22].
**A common threshold**	Three times the normal limit [1]	Three times the normal limit [1]

**Table 3:** Summary of the radiological tests utilized in acute pancreatitis.

Radiological Test	Advantages	Limitations	
US	Accessibility; Low expense; No exposure to radiation [31]; Allows diagnosis of acute biliary pancreatitis; Evaluates the condition of the biliary tract; Detects biliary stones in the CBD with high sensitivity and specificity [32,35].	Cannot be used to diagnose alcohol overuse as a main cause of AP; Unfavorable influence of intestinal gas occurring during ileus with bowel distension on quality of imaging; Adverse impact of food mass in the stomach on imaging of the pancreas—disruption of precision and completeness, creation of images falsely suggesting pancreatic tumors [32,35]; Poor quality, and therefore uncertain, diagnosis in the case of urgent management without proper preparation of the patient; Significant decrease in the sensitivity of detection of the gallstones localized in the infundibulum of the gallbladder or characterized by the diameter less than 3 mm [40].	
EUS	Minimal invasiveness; Lower complication rate in comparison to ERCP—allows the avoidance of complications associated with diagnostic ERCP [38,39,41]; Detection of the gallstones with higher sensitivity in comparison to US; Close proximity to the biliary system, allowing imaging of the gallbladder better than US and providing high-image resolution [39]; Improved spatial resolution in comparison to MRI and CT scan [41]; Regarded as a reasonable approach for assessment of patients with IAP/IRAP; Alternative to transabdominal ultrasound and tomographic examinations in the case of unsuccessful imaging of biliary calculi; Imaging of the entire gallbladder, pancreas, and biliary ductal system in AP in most cases [39].		
MRI	The non-invasive evaluation of pancreatic and biliary ducts, particularly the distal bile duct, which is hard to visualize by ultrasound; No exposure to radiation and subsequent adverse effects; No use of a contrast agent in non-enhanced images; Safe for the patients in the case of impossibility of receiving iodinated contrast material due to kidney failure or allergies; No premedication; No risk of developing complications; Possibility to use during acute attack of pancreatitis and cholangitis; Allows the visualization of the extraductal structures due to usage of standard T1-T2-weighted images; Non-enhanced MRI provides clear presentation of the area of necrosis; Visibly present local complications and stage the AP; Allows the imaging of even a small amount of fluid in mild pancreatitis [42]; Used to image a few fat or necrotic materials localized in a fluid-filled lesion and pancreatic duct system, which in turn allows the assessment of the duct integrity and whether collections surrounding the pancreas are in communication with pancreatic ducts [36,37]; Non-enhanced MRI provides more precise and reliable image in assessing the severity of AP in comparison with CT; Better soft-tissue contrast compared with CR; Non-enhanced MRI is better in diagnosis of mild AP compared with CT [36]; Allows the detection of pancreatic necrosis and complications of AP, such as abscesses, pseudocysts, or hemorrhage [28]; High sensitivity and specificity of MRCP in the diagnosis of biliary obstruction [46].	The diagnosis of AP is dependent on the occurrence of morphologic and peripancreatic changes [28]; High cost of MRCP, which limits its use in the diagnosis of gallstones [2,47].	
CT	Fast scans with high spatial resolution; Allows the imaging of the necrosis of the pancreas and local complications of AP; Enables the grading of the acuity of inflammation and the assessing of the severity of AP [36,37,48]; Provides essential information for percutaneous management [13]; High accuracy and sensitivity in diagnosing and providing the extent of the disease compared with US [48]; Used to exclude local complications and distinguish necrotizing acute pancreatitis and interstitial acute pancreatitis (more than 3–4 days from the onset of symptoms); Used in early diagnosis, in the case of the broad differential diagnosis that must be narrowed [2,49,50].	Difficulty to distinguish small quantity of necrotic or fat debris within one collection; Potential radiation risk in the case of numerous follow-up scans [36,37].	

**Table 4:** Balthazar CTSI-scoring.

Grade, Points	Characteristics
Grade A, 0 points	Normal pancreas.
Grade B, 1 point	Focal or diffuse enlargement of the pancreas (including contour irregularities, non-homogenous attenuation of the gland, dilation of the pancreatic duct, and foci of small fluid collections within the gland, as long as there was no evidence of peri-pancreatic disease.
Grade C, 2 points	Intrinsic pancreatic abnormalities associated with hazy streaky densities representing inflammatory changes in the peri-pancreatic fat.
Grade D, 3 points	Single ill-defined fluid collection (phlegmon).
Grade E, 4 points	Two or multiple poorly defined fluid collections or presence of gas in or adjacent to the pancreas.
**The presence and extent of necrosis**	**Points**
Necrosis absent	0 points
< 30% necrosis	2 points
30–50% necrosis	4 points
>50% necrosis	6 points
**Severity of AP**	**CTSI score**
Mild pancreatitis	0–3
Moderate pancreatitis	4–6
Severe pancreatitis	7–10

**Table 5:** Modified Mortele CTSI scoring.

Points	Characteristics
0 points	Normal pancreas
2 points	Intrinsic pancreatic abnormalities with or without inflammatory changes in peripancreatic fat.
4 points	Pancreatic or peripancreatic fluid collection or peripancreatic fat necrosis.
**The presence and extent of necrosis**	**Points**
Necrosis absent	0 points
<30% necrosis	2 points
>30% necrosis	4 points
**Severity of AP**	**Modified CTSI score**
Mild pancreatitis	0–2
Moderate pancreatitis	4–6
Severe pancreatitis	8–10

**Table 6:** Symptoms indicating the current or progressing organ dysfunction in the course of acute pancreatitis, the presence of which is a criterion for consideration of admission to a monitored unit.

Impaired Organ	Symptoms
Respiratory	Pao2/FiO2 ≤ 300 Respiratory rate > 20 breaths per min
Cardiovascular	Need for vasopressors in the case of non-fluid-responsive patients Hypotension, despite aggressive fluid resuscitation, defined as systolic blood pressure (sBP) < 90 mm Hg off inotropic support or drop of sBP > 40 pH < 7.3
Renal	4. Urine output < 0.5 mL/kg/h for ≥ 6 h 5. Increase of ≥ 26.5 μmol in serum creatinine over 48 h 6. ≥1.5-fold increase in serum creatinine over 7 days

## Data Availability

Please contact authors for data requests (Łukasz Olewnik—email address: lukasz.olewnik@umed.lodz.pl).
